# The Gut–Kidney Axis in Chronic Kidney Diseases

**DOI:** 10.3390/diagnostics15010021

**Published:** 2024-12-25

**Authors:** Kenji Tsuji, Naruhiko Uchida, Hiroyuki Nakanoh, Kazuhiko Fukushima, Soichiro Haraguchi, Shinji Kitamura, Jun Wada

**Affiliations:** 1 Department of Nephrology, Rheumatology, Endocrinology and Metabolism, Graduate School of Medicine, Dentistry and Pharmaceutical Sciences, Okayama University, 2-5-1 Shikata-cho, Okayama 700-8558, Japan; 2 Department of Nephrology, Aoe Clinic, Okayama 700-8607, Japan; 3 Department of Nursing Science, Faculty of Health and Welfare Science, Okayama Prefectural University, Okayama 719-1197, Japan

**Keywords:** gut–kidney axis, chronic kidney disease, uremic toxin, dysbiosis, gut microbiota

## Abstract

The gut–kidney axis represents the complex interactions between the gut microbiota and kidney, which significantly impact the progression of chronic kidney disease (CKD) and overall patient health. In CKD patients, imbalances in the gut microbiota promote the production of uremic toxins, such as indoxyl sulfate and p-cresyl sulfate, which impair renal function and contribute to systemic inflammation. Mechanisms like endotoxemia, immune activation and oxidative stress worsen renal damage by activating pro-inflammatory and oxidative pathways. Insights into these mechanisms highlight the impact of gut-derived metabolites, bacterial translocation, and immune response changes on kidney health, suggesting new potential approaches for CKD treatment. Clinical applications, such as dietary interventions, prebiotics, probiotics and fecal microbiota transplantation, are promising in adjusting the gut microbiota to alleviate CKD symptoms and slow disease progression. Current research highlights the clinical relevance of the gut–kidney axis, but further study is essential to clarify these mechanisms’ diagnostic biomarkers and optimize therapeutic interventions. This review emphasizes the importance of an integrated approach to CKD management, focusing on the gut microbiota as a therapeutic target to limit kidney injury.

## 1. Introduction

The gut–kidney axis reveals critical interactions between the gut microbiome and renal function that impact human health and disease. This communication pathway plays a central role in kidney health by mediating inflammation, uremic toxicity, and metabolic processes [1]. The gut microbiome, containing diverse microbes like short-chain fatty acid (SCFA)-producing bacteria from fiber fermentation, supports gut barrier integrity and reduces inflammation [2]. However, dysbiosis—an imbalance in the gut microbiota commonly seen in chronic kidney disease (CKD)—can disrupt these beneficial functions, leading to systemic toxicity and increased kidney burden [3].

Under physiological conditions, the gut–kidney axis maintains homeostasis by facilitating nutrient absorption, metabolite production, and immune tolerance. These interactions are essential for homeostasis both in the gut and kidneys [1]. In CKD, decreased kidney filtration restricts the excretion of uremic toxins, such as indoxyl sulfate and p-cresyl sulfate, leading to their accumulation in the bloodstream [4]. These toxins cause oxidative stress and inflammation in kidney tissues, accelerating CKD progression. Furthermore, dysbiosis and constipation in CKD patients can result in excess harmful metabolites, including ammonia and phenols, further stressing renal function and contributing to complications, such as cardiovascular disease [5]. Increased gut permeability, often referred to as “leaky gut”, is also associated with CKD, allowing endotoxins like lipopolysaccharides (LPS) to enter the bloodstream [6]. These endotoxins induce systemic inflammation, worsening nephritis and further stressing the kidneys [6]. Thus, the chronic inflammatory state seen in CKD patients is partly due to changes in the gut barrier and microbiota, emphasizing the role of the gut–kidney axis in disease progression.

Therapeutic strategies targeting the gut–kidney axis aim to restore microbial balance, reduce uremic toxins, and improve gut barrier function. Probiotics and prebiotics are increasingly studied for their potential to positively shift the gut microbiota, supporting beneficial bacteria and reducing harmful metabolites [7,8,9]. Dietary interventions, particularly high-fiber diets, promote SCFA production, which can strengthen gut barrier integrity and reduce systemic inflammation [10]. Treatments for constipation, fecal microbiota transplantation (FMT), and probiotics (bioactive compounds produced by bacteria) are being investigated for their ability to correct dysbiosis and lower toxin loads in CKD patients [3,4,5]. Although promising, significant gaps remain in our understanding of the specific mechanisms linking gut dysbiosis to renal pathology, as well as their long-term efficacy and safety. Additionally, studying the effects of specific microbial species and metabolites on kidney health could lead to precision therapies tailored to individual microbiomes. Addressing gut microbiota imbalances, reducing uremic toxins, and strengthening the gut barrier may help slow CKD progression. These approaches underscore the gut microbiota as a pivotal therapeutic target, not only for managing CKD but also for mitigating systemic complications, such as cardiovascular diseases [4]. This review seeks to address these gaps by synthesizing recent advancements in the field and highlighting emerging areas of research, focusing on the gut microbiota as a therapeutic target to limit CKD progression.

## 2. Gut Microbiome Under Physiological Condition

### 2.1. Physiological Effects of Gut Microbiota

The gut microbiome consists of a variety of bacteria, fungi and viruses. Approximately 100 trillion bacteria inhabit the human intestines, forming the gut microbiota [11]. Key gut microbiomes include *Firmicutes*, *Bacteroides*, *Ruminococcus* and *Bifidobacterium* [12]. The gut microbiota exerts significant physiological effects on the human body, influencing metabolic, immune and neurobehavioral processes [13]. This vast microbial community, mainly bacterial, exists symbiotically within the digestive tract, where its composition and diversity have profound implications for human health. These bacteria produce essential metabolites, such as SCFAs and vitamins, which regulate gut barrier integrity [14]. Changes in microbiome composition, as seen in CKD, can substantially affect the kidneys, with pathogenic strains often outnumbering beneficial bacteria in CKD [15].

### 2.2. Metabolic Effects and Integrity and Function of the Gut

The gut microbiota plays a crucial role in digestion and energy homeostasis [16]. It can break down indigestible plant polysaccharides and resistant starch, thus facilitating the absorption of complex carbohydrates. In a healthy gut, probiotics continuously proliferate and smoothly synthesize vitamins [17]. By breaking down dietary fiber and producing SCFAs, such as acetate, propionate and butyrate, the microbiota assists in nutrient absorption and energy extraction [18]. Butyrate, in particular, serves as an energy source for colon cells, supporting gut health and maintaining the gut barrier [19]. SCFAs also influence metabolic pathways, such as glucose regulation and lipid metabolism, by acting on peripheral tissues through signaling pathways that affect insulin sensitivity and lipid processing [20]. Dysbiosis (microbial imbalance) may contribute to metabolic disorders, including obesity, insulin resistance and type 2 diabetes [21]. The gut microbiota also communicates bidirectionally with the brain, affecting neurobehavioral function via the gut–brain axis. Microbial metabolites, such as SCFAs and neurotransmitter precursors (e.g., serotonin), influence mood, stress response and cognitive function by modulating the vagus nerve, immune pathways and hormonal signaling [22]. For example, SCFAs can cross the blood–brain barrier, impacting brain function and behavior [23]. Emerging research highlights correlations between gut microbiota imbalance and neuropsychiatric disorders like depression, anxiety and autism spectrum disorders, suggesting potential mental health benefits in restoring a healthy microbiome [24]. Additionally, the gut microbiota aids in maintaining gut barrier integrity by promoting tight junction proteins that prevent pathogen invasion. Microbe-derived SCFAs, especially butyrate, strengthen the gut barrier and reduce inflammation, protecting against pathogens and lowering the risk of systemic infections [25]. Dysbiosis weakens this barrier, increasing gut permeability and causing a condition known as “leaky gut”, where microbial endotoxins enter the bloodstream [26], potentially leading to systemic inflammation associated with chronic diseases, such as cardiovascular disease and type 2 diabetes.

### 2.3. Immunological Effects

The gut microbiota is central to immune system development, distinguishing between beneficial and harmful microbes [27]. Early exposure to microbes influences immune tolerance and shapes immune responses. Specific bacterial species, such as *Bacteroides* and *Lactobacillus genera*, promote the generation of regulatory T cells (Tregs), which play a role in controlling inflammation and preventing autoimmune diseases [28,29]. Furthermore, the microbiota interacts with gut-associated lymphoid tissue (GALT), contributing to the secretion of immunoglobulin A (IgA), which protects against pathogens [30]. Dysbiosis in the gut microbiota is associated with chronic inflammatory diseases, including inflammatory bowel disease (IBD), rheumatoid arthritis, and allergies [31,32]. SCFAs exert multiple beneficial effects on the immune system. SCFAs promote the differentiation of Tregs in the gut through histone deacetylase (HDAC) inhibition and G protein-coupled receptor (GPRs) activation, such as GPR43, GPR41 and GPR109a [4,33,34]. Inhibition of HDACs leads to suppression of the nuclear factor-kappa beta (NF-κB) in the mucosal immune system, thereby influencing transcription of inflammatory-associated genes, including interleukin-6 (IL-6) and tumor necrosis factor-alpha (TNF-α). Additionally, activation of GPRs could regulate inflammatory processes partly through suppressing the expression of NF-κB and promoting the differentiation of colonic Tregs [4].

## 3. Gut Microbiome in Kidney Diseases: Dysbiosis in CKD, Microbial Metabolites and Toxins

### 3.1. Dysbiosis in CKD

**Figure 1:**
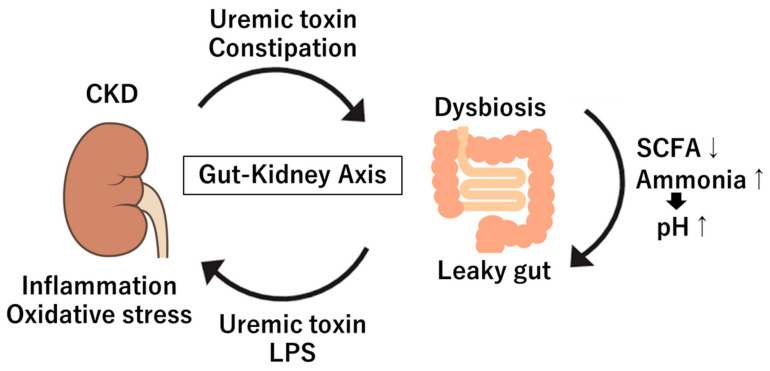
Gut–kidney axis. CKD: chronic kidney disease; LPS: lipopolysaccharides; SCFA: short-chain fatty acid; ↑: increase; ↓: decrease.

In CKD, dysbiosis is characterized by a decrease in beneficial bacteria, such as *Lactobacillus*, *Prevotella* and *Bifidobacteria*, alongside an increase in pathogenic or opportunistic bacteria, including *Proteobacteria* and *Enterococcus* [5,35,36,37,38,39,40]. The mechanisms underlying dysbiosis in CKD are not fully understood; however, several factors are implicated. These include the accumulation of uremic toxins due to renal dysfunction, metabolic acidosis, the effects of chelating agents such as oral iron supplements, potassium and phosphorus used in CKD treatment, as well as impaired intestinal function, reduced dietary fiber intake, and constipation frequently seen in CKD patients [5,41]. Such changes in the gut microbiota are influenced by dietary factors (e.g., reduced fiber intake), medications and the uremic environment itself [41]. As renal function declines, uremic toxins such as urea accumulate in the bloodstream, potentially infiltrating the gut and compromising the gut barrier [42]. This disruption allows harmful metabolites to enter systemic circulation, triggering systemic inflammation and accelerating renal injury [42] (Figure 1). Additionally, CKD patients commonly exhibit “leaky gut” conditions due to increased intestinal permeability, which is exacerbated by uremia, intestinal edema and ischemic changes in the gut [6]. This is associated with reduced expression of tight junction proteins in the intestinal epithelium (ZO-1, claudin, and occludin), allowing endotoxins, including LPS, to migrate into the bloodstream [43,44]. Endotoxemia subsequently provokes immune responses, stimulating pro-inflammatory cytokines (such as IL-6 and TNF-α) and perpetuating a chronic inflammatory state that may further degrade renal function [43,44]. Indeed, CKD patients show higher levels of circulating endotoxins than healthy individuals, suggesting compromised gut barrier function [45]. The leaky gut in CKD perpetuates a feedback loop where inflammation further disturbs the gut microbiome, creating a vicious cycle that exacerbates kidney disease progression.

### 3.2. Microbial Metabolites and Toxins

**Figure 2:**
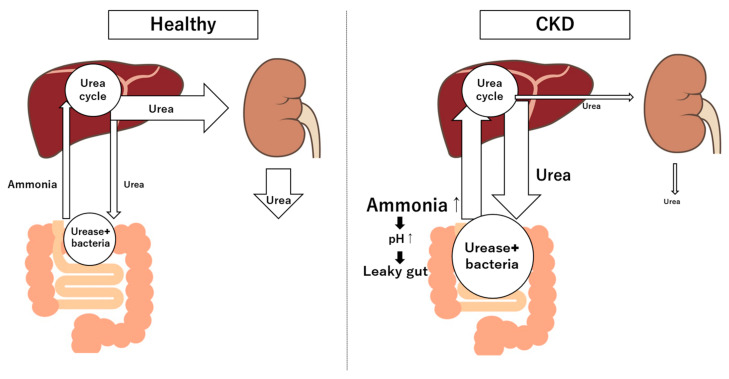
Urea and ammonia metabolism in health and CKD. CKD: chronic kidney disease.

Gut-derived metabolites can have both beneficial and harmful effects on the kidney. SCFAs, for instance, help maintain the gut barrier function [46]. On the other hand, toxins such as ammonia and phenols—produced during protein fermentation by urease-producing gut bacteria—promote systemic inflammation and damage to the kidneys [47]. This metabolic imbalance creates a cycle of toxicity, as impaired kidneys reduce toxin clearance, intensifying renal and systemic injury. Harmful substances, such as ammonia, amines, thiols, phenols, and indoles, are produced by proteolytic gut bacteria, such as *Bacteroides* and *Clostridium*, which are increased in CKD [48]. Ammonia and urea are byproducts of protein catabolism, and the urea is converted back into ammonia by gut bacteria in the colon via urease [49] (Figure 2). In CKD, the accumulation of urea in the blood leads to an increased influx of urea into the gastrointestinal tract, which increases ammonia production [50]. Elevated ammonia levels disrupt the local environment by raising pH, which can weaken the gut barrier and lead to epithelial cell damage [50]. Ammonia has been found to affect the integrity of tight junction proteins like ZO-1 and occludin in the intestinal lining [51]. This disruption in tight junctions compromises the gut barrier, potentially leading to a “leaky gut” state. Additionally, epithelial injury from high ammonia may trigger a cascade of immune responses, further increasing inflammatory cytokine production (e.g., IL-6, TNF-α) and aggravating chronic inflammation associated with CKD. Furthermore, an altered gut environment due to ammonia may promote the growth of urease-producing proteolytic bacteria, which produce additional toxins harmful to the kidneys and other tissues. This can create a vicious cycle where ammonia not only directly damages intestinal epithelial cells but also promotes systemic inflammation that exacerbates kidney disease progression. Uremic toxins, including indoxyl sulfate, p-cresyl sulfate, and trimethylamine N-oxide (TMAO), which significantly contribute to CKD progression and vascular complications, are derived from dietary components and metabolized by gut bacteria [52,53,54]. These uremic toxins exist in the bloodstream and are primarily bound to proteins, such as albumin, rather than in a free state [55]. This protein-binding limits their removal during dialysis and contributes to their accumulation and toxicity in CKD. Protein fermentation in the gut generates various metabolites that greatly impact kidney homeostasis [56]. A protein-rich diet and uremic conditions can lead to the overgrowth of protein-fermenting bacteria, producing toxic byproducts like indoxyl sulfate, p-cresyl sulfate, and ammonia [57,58]. These protein fermentation metabolites contribute to systemic toxicity, promoting the progression of CKD and associated cardiovascular complications.

#### 3.2.1. Indoxyl Sulfate

Indoxyl sulfate originates from the bacterial metabolism of tryptophan, an amino acid in protein-rich foods [59]. After being absorbed, it undergoes hepatic modification and becomes indoxyl sulfate, which is excreted by the kidneys [52]. In CKD, reduced kidney clearance allows indoxyl sulfate to accumulate in the bloodstream, acting as a uremic toxin. It promotes oxidative stress and inflammation in kidney tissues by stimulating reactive oxygen species (ROS) production and enhancing pro-fibrotic gene expression, accelerating tubular injury and interstitial fibrosis, both of which contribute to CKD progression [52]. It is also reported that indoxyl sulfate affects cardiovascular tissues, causing a greater risk to CKD patients who are already prone to cardiovascular complications [60].

#### 3.2.2. P-Cresyl Sulfate

Similar to indoxyl sulfate, p-cresyl sulfate is a gut bacteria-derived metabolite from amino acids, specifically tyrosine and phenylalanine [59]. After undergoing sulfation in the liver, it circulates in the bloodstream, accumulating under renal dysfunction. P-cresyl sulfate exerts pro-inflammatory effects on both the kidneys and vascular system, contributing to endothelial dysfunction and promoting atherosclerosis [52,61]. In kidney tissues, it induces oxidative stress and inflammation, impairing kidney tubular cells and accelerating disease progression [61]. Elevated p-cresyl sulfate levels correlate with higher mortality rates in CKD patients [62], underscoring its role as a significant uremic toxin. In addition, it is reported that p-cresyl sulfate and indoxyl sulfate contribute to vascular calcification through mechanisms involving oxidative stress, inflammation, and the promotion of osteogenic differentiation in vascular smooth muscle cells [55].

#### 3.2.3. Trimethylamine-N-Oxide (TMAO)

TMAO is produced from dietary sources, especially from choline and carnitine intake found in red meat and eggs [53,63]. Gut bacteria convert these compounds into trimethylamine, which is then oxidized in the liver to form TMAO [53,63]. Elevated TMAO levels are associated with increased cardiovascular risk, a leading cause of death among CKD patients [64]. TMAO has been shown to promote vascular inflammation and enhance platelet aggregation and macrophage activation, further exacerbating cardiovascular complications [53]. TMAO may also worsen renal damage by influencing inflammatory pathways [64].

## 4. Mechanisms of Interaction Between Gut and Kidney

### 4.1. Systemic Inflammation and Immune Activation

CKD is associated with systemic inflammation, and gut dysbiosis plays an important role in this process. A decrease in beneficial bacteria and an overgrowth of pathogenic bacteria increase exposure to endotoxins and other harmful molecules, which activate the immune system [4,5]. Specifically, harmful bacteria produce metabolites, such as LPS, that cross the intestinal barrier due to increased permeability [6], the “leaky gut”. When LPS enters the circulation, it activates immune cells to produce pro-inflammatory cytokines, such as IL-6 and TNF-α, which play a role in promoting CKD progression and vascular inflammation [5,6]. This immune activation not only impacts renal function but also raises the risk of cardiovascular disease, as inflammatory mediators damage blood vessels and promote plaque formation [5]. Cytokine-induced inflammation directly causes kidney injury, exacerbating glomerulosclerosis and fibrosis.

### 4.2. Endotoxemia and Kidney Inflammation and Oxidative Stress

The presence of endotoxins, such as LPS, in the bloodstream is a key factor in kidney inflammation [65,66]. In CKD, the gut barrier is often compromised by both gut dysbiosis and uremic toxins, increasing intestinal permeability [50]. As LPS and other microbial products leak into the bloodstream, they trigger an inflammatory response that has significant effects on renal function [43,44]. When endotoxins reach the kidneys, they activate Toll-like receptors (TLRs) on renal cells, initiating a cascade of pro-inflammatory and oxidative stress responses [67]. These include the upregulation of cytokines and chemokines, leading to increased immune cell infiltration in the kidneys. This process is further aggravated by oxidative stress, a phenomenon where excess ROS damages cellular components and intensifies inflammation [67]. Oxidative stress, partially induced by gut-derived toxins, can directly injure renal tubular cells, promoting fibrosis and impairing renal function [68]. The combined effects of endotoxemia and oxidative stress establish a harmful feedback loop, wherein chronic inflammation and oxidative stress gradually worsen renal function, exacerbating the clinical symptoms of CKD.

### 4.3. Dietary Carbohydrates Fermentation

The fermentation of dietary carbohydrates in the gut primarily produces SCFAs such as acetate, propionate and butyrate, which play a protective role in kidney health. SCFAs are known to have anti-inflammatory effects and help maintain intestinal barrier integrity [46]. In CKD, however, gut dysbiosis reduces SCFA-producing bacteria (*Bifidobacterium*, *Lactobacillus*), weakening this protective effect [69]. The decreased intake of dietary fiber often observed in CKD also reduces SCFA production, potentially allowing pathogenic bacteria to proliferate [70]. Reduced SCFA levels weaken the gut barrier, increasing permeability and endotoxemia, which subsequently cause inflammation and oxidative stress in the kidneys [71,72]. This breakdown of tight junctions is associated with endotoxemia from gut-derived sources, elevated blood CRP levels, and increased mortality rates, with gut microbiome-derived DNA from species, including *Klebsiella* spp., *Proteus* spp., *Escherichia* spp., *Enterobacter* spp. and *Pseudomonas* spp. detected in the bloodstream of approximately 20% of end-stage renal disease (ESRD) patients [73,74]. Furthermore, low SCFA levels impair inflammation regulation, worsening immune activation [71]. This pro-inflammatory state, exacerbated by endotoxemia and oxidative stress, contributes to CKD progression.

### 4.4. Advanced Glycation Products

Advanced glycation end products (AGEs) are closely associated with the gut–kidney axis through systemic inflammation. AGEs are compounds formed through the reaction of sugars with proteins or lipids, a process that can occur endogenously or through dietary intake, especially from high-temperature cooking methods, such as frying or grilling [75]. AGEs accumulate in CKD due to reduced renal clearance, and they can also be absorbed from certain foods, especially processed foods [75]. AGEs can contribute to renal damage through multiple pathways [76]. When AGEs bind to their receptor (RAGE), they activate pro-inflammatory pathways and promote oxidative stress, exacerbating renal inflammation and fibrosis [75,76]. AGE accumulation in the gut increases permeability, allowing LPS to enter the bloodstream. This process damages endothelial cells, leading to vascular stiffness and increased blood pressure [76]. It is also reported that AGEs contribute to glomerular sclerosis, one of the primary pathological features of CKD [75,77]. High-AGE diets have been shown to elevate systemic levels of AGEs [75,78], leading to more significant kidney damage in CKD patients. Reducing dietary AGE intake, along with minimizing the production of endogenous AGEs, has potential therapeutic value in managing CKD progression.

### 4.5. Ketone Bodies

Ketone bodies (acetoacetate, β-hydroxybutyrate, and acetone) are energy metabolites produced through fatty acid metabolism, primarily generated under conditions of starvation or low carbohydrate intake. Ketone body metabolism in the kidney plays a critical role, serving as an energy source, reducing oxidative stress, and exerting anti-inflammatory effects [79,80]. In the context of the gut–kidney axis, ketone body production can modulate the composition of gut microbiota, promoting the growth of beneficial SCFA-producing bacteria. This shift in microbial balance improves gut barrier integrity. High-fat, low-carbohydrate diets, which stimulate endogenous ketone body production, have shown potential renal protective effects [81,82]. However, the long-term safety of ketogenic diets in patients with CKD requires further investigation, partly due to the potential of high-fat diets to exacerbate metabolic acidosis by increasing dietary acid load and endogenous acid production [82].

## 5. Clinical Implications and Therapeutic Approaches

**Figure 3:**
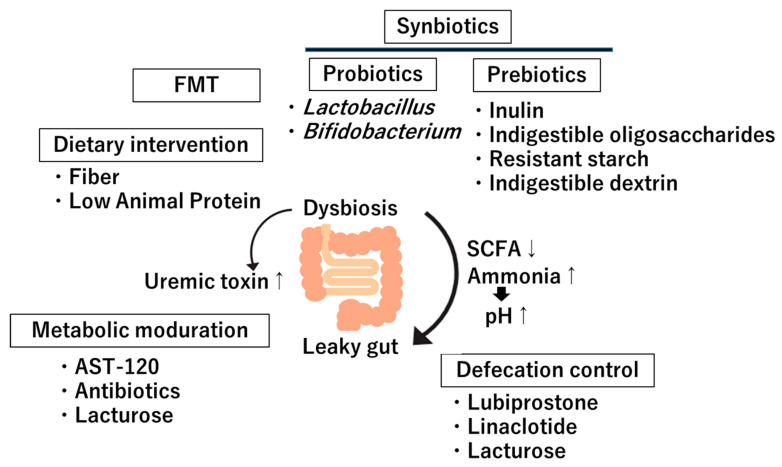
Therapeutic Approaches against dysbiotics in the gut–kidney axis. FMT: Fecal Microbiota Transplantation; SCFA: short-chain fatty acid; ↑: increase; ↓: decrease.

The gut–kidney axis has emerged as a significant area of study due to the growing understanding of the microbiota’s role in influencing kidney health. Interventions targeting this axis are increasingly considered for CKD management. These strategies encompass dietary interventions, probiotics and prebiotics, FMT, metabolite modulation, and enhancing defecation (Figure 3 and Table 1).

### 5.1. Dietary Intervention

Dietary interventions are foundational for CKD management, as diet directly influences the composition and metabolic activity of the gut microbiota [84]. A diet high in fiber and low in animal protein promotes the growth of beneficial bacteria that produce SCFAs [83,84], which are associated with reduced production of uremic toxins. SCFAs have anti-inflammatory properties that strengthen the gut barrier and reduce gut-derived inflammation impacting the kidneys [2]. Conversely, diets high in red or processed meats increase the production of indoxyl sulfate and p-cresyl sulfate, which are metabolites linked to renal injury [85,86]. Plant-based and Mediterranean diets are particularly beneficial as they supply prebiotic fibers that promote beneficial bacteria, lower uremic toxin levels, and enhance overall gut and kidney health [87]. Regular inclusion of these dietary modifications can reduce systemic inflammation and oxidative stress, potentially slowing CKD progression. Dietary fiber alleviated the gut microbiota, elevating the level of Bacteroides acidifaciens [99]. A high-fiber diet not only restores the gut microbiota and metabolome in plasma, cecum, and urine but may also slow CKD progression [100]. These effects may be mediated by the restoration of tight junctions in the gut epithelium, attenuation of oxidative stress, and reduction in inflammation and fibrosis partly via an increase in SCFA-producing bacteria [101,102].

### 5.2. Probiotics

Probiotics, often referred to as “good bacteria”, are live microorganisms that offer health benefits by improving the balance of the gut microbiota [103]. Common strains include *Lactobacillus* and *Bifidobacterium*, both known to support gut health and alleviate dysbiosis in CKD patients [104]. Probiotics compete with harmful bacteria for resources, reduce toxin levels, and promote gut barrier integrity [105,106]. Studies have demonstrated that probiotic supplementation in CKD patients can reduce levels of uremic toxins, potentially slowing the progression of renal dysfunction [107,108,109,110,111,112]. For instance, supplementation with *Lactobacillus acidophilus* and *Bifidobacterium* has been associated with decreased inflammatory markers in CKD, indicating beneficial effects beyond the gastrointestinal tract [88]. Probiotics may also modulate immune responses, reducing pro-inflammatory cytokine production, such as IL-6 and TNF-α, which lowers systemic inflammation [105,106]. Additionally, using probiotics in CKD may have cardiovascular benefits, which is a crucial consideration since CKD often coexists with cardiovascular issues [103,113].

### 5.3. Prebiotics

Prebiotics are indigestible food components that stimulate the growth and activity of beneficial bacteria in the gut [114]. Examples of prebiotics include fibers, such as inulin, fructo-oligosaccharides, resistant starch, indigestible dextrin (such as resistant dextrin) and galacto-oligosaccharides, which ferment in the gut to produce SCFAs [89,90,91]. As described, these SCFAs play a crucial role in maintaining gut barrier function by promoting immune tolerance and reducing systemic inflammation. In the context of CKD, where gut permeability is often compromised, prebiotics help strengthen the gut barrier and reduce the translocation of endotoxins into the bloodstream, which can exacerbate kidney inflammation [90,91,107]. Prebiotic supplementation has been shown to be reno-protective in CKD by reducing gut-derived uremic toxins [115,116]. By promoting the growth of SCFA-producing bacteria, prebiotics indirectly lower levels of indoxyl sulfate and p-cresyl sulfate. SCFAs also protect the kidney by inhibiting pro-inflammatory pathways and improving glucose and lipid metabolism, both essential for managing the metabolic disorders associated with CKD.

Clinical studies evaluating prebiotics in CKD patients show promising results in improving gut microbiota composition and reducing inflammatory markers and uremic toxins [89,90,91,115,116]. For example, inulin-type prebiotics are associated with increased levels of beneficial *Bifidobacterium* and *Faecalibacterium prausnitzii* [117], known for their anti-inflammatory effects. Galacto-oligosaccharides have been found to increase beneficial gut bacteria while reducing pathogenic bacteria [118,119]. Combining probiotics and prebiotics as synbiotics (a strategy that utilizes both in parallel) may have even greater potential in modulating the gut microbiota and slowing kidney disease progression [90,91]. Synbiotic formulations are thought to enhance the survival and efficacy of probiotics by providing a nutritional source (prebiotics), more effectively promoting colonization in the gut. In summary, probiotics and prebiotics represent promising, non-invasive interventions for CKD management. They offer potential benefits through gut microbiota modulation, reduction of uremic toxin levels, and improved gut barrier integrity. However, further research is necessary to refine dosage guidelines, optimize strain selection, and understand the long-term impacts on CKD progression and patient outcomes.

### 5.4. Fecal Microbiota Transplantation (FMT)

FMT involves transferring fecal material from a healthy donor to a recipient, effectively replacing a dysbiotic gut microbiome with a balanced one [120]. Although still in the experimental stages for CKD, FMT has shown promising results in inflammatory and metabolic disorders [121,122], suggesting potential benefits in kidney diseases. The rationale is that FMT can restore a healthy microbiota composition, reducing endotoxemia and systemic inflammation, which are key contributors to CKD [123,124]. In preclinical studies, FMT has been shown to reduce renal injury and inflammation by normalizing gut barrier integrity and reducing levels of pro-inflammatory metabolites [92]. However, FMT carries risks, such as the transmission of infections or unforeseen immune reactions, so further research is required to assess safety, efficacy, and the best protocols for its application in CKD [5]. Nonetheless, as a therapeutic tool, FMT represents an innovative approach with the potential to significantly modify the gut–kidney axis and alleviate CKD symptoms.

### 5.5. Metabolites Modulation

The modulation of gut-derived metabolites, particularly uremic toxins, is an important approach to addressing gut–kidney axis dysfunction. Targeting specific toxins, including indoxyl sulfate and p-cresyl sulfate, can mitigate adverse effects on the kidneys. This can be achieved through dietary interventions, prebiotics and adsorbents that capture and reduce the absorption of harmful metabolites. Pharmaceutical interventions have also shown promise. For example, AST-120 is an oral adsorbent that binds indole and other indoxyl sulfate precursors in the gut to reduce uremic toxins [125]. Although AST-120 is not widely accepted in all medical guidelines, randomized controlled trials in Japan involving pre-dialysis CKD patients (CAP-KD trial) demonstrated a significantly improved rate of renal function decline with AST-120 administration [93,94]. Its effectiveness in slowing CKD progression by reducing indoxyl sulfate levels has been validated. However, a large-scale study in Western countries (the EPPIC trial) found no significant CKD progression delay with AST-120 compared to a placebo [126], potentially due to adherence issues and adverse impacts on the gut environment. Further research may identify other compounds or pharmacologic agents capable of modulating gut-derived toxins and providing additional therapeutic options for kidney disease patients. Recent studies reported that administering the oral antibiotic vancomycin to remove gut microbiota in ESRD patients significantly reduced plasma concentrations of indoxyl sulfate and p-cresyl sulfate [95]. However, it is crucial to minimize overall exposure to vancomycin to reduce the risk of developing vancomycin-resistant enterococci (VRE) [127]. On the other hand, germ-free mice with ischemia-reperfusion (I/R)-induced acute kidney injury (AKI) and CKD models demonstrated more severe renal damage than mice with a symbiotic microbiota [128], possibly due to the beneficial effects of SCFAs. A sodium-glucose co-transporter (SGLT)-2 inhibitor, canagliflozin, in renal failure mice, changed the gut microbiota composition and reduced gut-derived uremic toxins by increasing glucose delivery to the distal intestine through SGLT-1 inhibition [96]. SGLT-2 inhibitors are widely used to improve kidney outcomes [129], while the mechanisms underlying their reno-protective effects are not fully understood. These mechanisms may involve the gut–kidney axis, partly through the enhancement of ketone body production by shifting energy metabolism [79].

### 5.6. Defecation Modulation

Regular bowel movements are crucial for gut–kidney health, as they affect the retention of gut-derived toxins [130]. CKD patients, particularly those on maintenance dialysis, experience a high prevalence of constipation [130,131]. Mechanisms contributing to constipation include impaired blood flow to the intestines, neurological issues affecting intestinal peristalsis, side effects of certain medications (e.g., ion-exchange resins or oral iron supplements), dietary restrictions (e.g., potassium restrictions reducing fiber intake) and changes in the gut microbiota [130,132]. Constipation in CKD can prolong colonic transit time, increase the retention of intestinal contents, promote putrefaction reactions, and potentially worsen CKD. An epidemiological study in non-CKD patients indicated that those with constipation had a higher rate of progression to CKD and increased risk of ESRD compared to those without constipation [41]. Therefore, bowel regulation interventions, such as fiber supplementation, laxatives, and stool softeners, may serve as an effective, indirect approach to reducing gut–kidney axis dysfunction. Increased intake of dietary fiber and water encourages regular bowel movements, allowing uremic toxins to be excreted before entering systemic circulation [133]. Among laxatives, lubiprostone activates chloride channels in intestinal epithelial cells, increasing intestinal fluid secretion and promoting content movement, providing a laxative effect [134]. Studies in renal failure mice show that lubiprostone improves the altered gut environment associated with renal failure, reduces the accumulation of gut-derived blood uremic toxins, and slows CKD progression [38]. It has also been shown to increase bacteria typically reduced in CKD, such as *Lactobacillus* and *Prevotella* species. Linaclotide has shown effects in renal failure mice, reducing TMAO levels and alleviating renal damage and myocardial fibrosis [97]. Lactulose also slows the progression of CKD in renal failure mice [98]. Importantly, lactulose may act not only as defecation modulation but also as prebiotic and metabolic modulation. As a prebiotic, lactulose resists digestion in the upper gastrointestinal tract, reaching the colon where it is fermented by beneficial bacteria, such as *Bifidobacteria* and *Lactobacillus* [135,136], which outcompete ammonia-producing bacteria and enhance SCFA production. Furthermore, as modulation of ammonia metabolism, SCFAs acidify the gut lumen, leading to an increase in ammonium ions and a decrease in freely absorbable ammonia [137]. This modulation of ammonia metabolism may reduce serum ammonia levels and help limit the accumulation of uremic toxins that are toxic to the kidneys. Indeed, lactulose significantly decreased urea levels in the clinical prospective study [138]. In addition, a randomized clinical trial revealed that lactulose increased *Bifidobacteria* and *Lactobacillus* and decreased serum creatinine levels, suggesting the reno-protective effect presumably via modulation of the microbiome [139].

Each of these therapeutic approaches—dietary interventions, probiotics and prebiotics, FMT, metabolite modulation and bowel movement regulation—holds promise in mitigating the CKD progression via the gut–kidney axis. Together, they represent a multifaceted approach to managing CKD progression, reducing inflammation, systemic toxicity and oxidative stress. Further research is essential for specific interventions, especially FMT and metabolite-focused treatments, but the gut–kidney axis remains a promising therapeutic target in CKD management. These strategies collectively emphasize the potential of modulating gut health to improve kidney outcomes, a potential that continues to expand with advances in microbiome and nephrology research.

## 6. Conclusions

The gut–kidney axis involves complex interactions mediated by systemic inflammation, endotoxemia, oxidative stress, and metabolites derived from protein and carbohydrate fermentation, as well as dietary AGEs [3,4,5,6]. Each of these pathways contributes to a cycle of inflammation, immune activation, and oxidative stress, creating a damaging feedback loop that accelerates CKD progression. Addressing these mechanisms, potentially through dietary interventions, prebiotics, and therapeutics targeting gut-derived toxins, may offer new pathways to slow kidney disease progression and improve patient outcomes. This comprehensive understanding of the gut–kidney axis highlights the importance of gut microbiota management in CKD and underscores the need for future research focused on targeted treatments to interrupt these pathological processes. The gut–kidney axis is part of a larger network, sometimes called the gut–kidney–cardiovascular (heart) axis, due to the overlapping effects that dysbiosis and gut-derived toxins have on both kidney and cardiovascular health [5]. The gut–kidney axis significantly impacts cardiovascular disease associated with CKD. Dysbiosis and uremic toxins in the gut can contribute to increased rates of atherosclerosis, hypertension, and heart disease in CKD patients. The accumulation of indoxyl sulfate, p-cresyl sulfate and TMAO contributes to a pro-inflammatory and pro-oxidative state that aggravates kidney and cardiovascular diseases. This relationship highlights the importance of managing kidney health and cardiovascular risk factors through a gut-focused approach.

There remain gaps in understanding species-specific microbiota roles and causative mechanisms within the gut–kidney axis. Further research on microbial signaling pathways linking gut dysbiosis, systemic inflammation, and kidney fibrosis, as well as the role of individual microbial species and longitudinal studies tracking microbiome changes, are crucial to refining therapeutic strategies. Next-generation probiotics, designed to target specific bacterial strains and precision microbiome-modulation techniques, represent future treatment avenues. Individualized therapies based on a patient’s unique microbiome profile may optimize gut–kidney health by reducing toxin levels and reinforcing the gut barrier. In addition, in the context of an activated urea cycle in CKD, an intervention targeting gut ammonia production or adsorption could potentially reduce uremic toxin levels. This approach may include the use of urease inhibitors or specialized ammonia adsorbents.

In summary, the gut–kidney axis represents a promising target for managing CKD and related complications. By addressing gut dysbiosis, reducing uremic toxins, and improving gut barrier integrity, therapeutic interventions aimed at the microbiome hold the potential to slow CKD progression and enhance patient outcomes. Further research is essential for developing microbiome-based treatments that could transform kidney disease management.

## Figures and Tables

**Table 1:** Potential treatments targeting the gut–kidney axis.

Treatments	Targeting Method	Outcome	Ref.
Diet Intervention	High-fiber diet	Increased bacteria-producing SCFAs	[83,84]
	Low red/processed meats	Reduced uremic toxins	[85,86]
	Plant-based diet	Reduced uremic toxins	[87]
Probiotics	Lactobacillus Bifidobacterium	Reduced toxin levels Promotion of gut barrier integrity Decreased inflammatory markers	[88]
Prebiotics	Inulin Fructo-oligosaccharides, Resistant starch Indigestible dextrin Galacto-oligosaccharides	Increased beneficial bacteria Increased SCFAs	[89,90,91]
FMT	FMT	Promotion of gut barrier integrity Reducing pro-inflammatory metabolites	[92]
Metabolic Modulation	AST-120	Reduced indoxyl sulfate	[93,94]
	Vancomycin	Reduced indoxyl sulfate and p-cresyl sulfate	[95]
	SGLT-2 inhibitor	Reduced gut-derived uremic toxins	[96]
Defecation Modulation	Lubiprostone	Reduced gut-derived uremic toxins	[38]
	Linaclotide	Reduced TMAO levels	[97]
	Lactulose	Reduced indoxyl sulfate	[98]

SCFAs, short-chain fatty acid; FMT, Fecal Microbiota Transplantation; SGLT-2, sodium-glucose co-transporter-2; TMAO, trimethylamine N-oxide.

## Data Availability

No datasets were generated during and/or analyzed during the current study.
